# The genome of the endophytic bacterium *H. frisingense* GSF30^T^ identifies diverse strategies in the *Herbaspirillum* genus to interact with plants

**DOI:** 10.3389/fmicb.2013.00168

**Published:** 2013-06-27

**Authors:** Daniel Straub, Michael Rothballer, Anton Hartmann, Uwe Ludewig

**Affiliations:** ^1^Institut für Kulturpflanzenwissenschaften, Ernährungsphysiologie der Kulturpflanzen (340h), Universität HohenheimStuttgart, Germany; ^2^Helmholtz Zentrum München, National Research Center for Environmental Health, Research Unit Microbe-Plant InteractionsNeuherberg, Germany

**Keywords:** microbe, diazotroph, nitrogen fixation, plant associated bacteria, plant growth promoting bacteria

## Abstract

The diazotrophic, bacterial endophyte *Herbaspirillum frisingense* GSF30^T^ has been identified in biomass grasses grown in temperate climate, including the highly nitrogen-efficient grass *Miscanthus*. Its genome was annotated and compared with related *Herbaspirillum* species from diverse habitats, including *H. seropedicae*, and further well-characterized endophytes. The analysis revealed that *Herbaspirillum frisingense* lacks a type III secretion system that is present in some related *Herbaspirillum* grass endophytes. Together with the lack of components of the type II secretion system, the genomic inventory indicates distinct interaction scenarios of endophytic *Herbaspirillum* strains with plants. Differences in respiration, carbon, nitrogen and cell wall metabolism among *Herbaspirillum* isolates partially correlate with their different habitats. *Herbaspirillum frisingense* is closely related to strains isolated from the rhizosphere of *phragmites* and from well water, but these lack nitrogen fixation and metabolism genes. Within grass endophytes, the high diversity in their genomic inventory suggests that even individual plant species provide distinct, highly diverse metabolic niches for successful endophyte-plant associations.

## Introduction

Many gramineous species maintain a close association with endophytic bacteria that are often beneficial for plant growth and health (Reinhold-Hurek and Hurek, 1998). Their considerable ecologic importance and agronomic potential is best documented in warm tropical and subtropical climates (Reinhold-Hurek and Hurek, 1998). Plant growth promoting bacteria are often considered as a cost efficient and ecological alternative to improve crop growth on low-nutrient soils (Sturz et al., 2000) and may gain further interest for future large-scale biomass production on marginal land with low-input grasses (Heaton et al., 2008).

*Herbaspirillum frisingense* belongs to the β-proteobacteria and is a close relative of *Herbaspirillum seropedicae* SmR1 (*HsSmR1*) and *Herbaspirillum rubrisubalbicans* (*HrM1*), which are both common in tropical and subtropical soils and endophytically colonize various grasses (Monteiro et al., 2012b). Endophytes are referred to here as microorganisms (bacteria) that have low soil competence and spend most of their life cycle within the plant, mostly without causing symptoms of plant damage. Beneficial associations of *HsSmR1* and *HrM1* with sorghum, sugar cane, rice, and maize have been reported, but *HrM1* causes red stripe disease on some sorghum varieties and can cause mottled stripe disease on sugarcane. Other isolates of *H*. *seropedicae* from rice (*HsOs34*, *HsOs45*) induced disease symptoms (Ye et al., 2012; Zhu et al., 2012). So far, plant growth promoting action, but no disease symptoms, were identified for *H. frisingense* (Straub et al., unpublished observation), which was originally isolated from the perennial C4-fiber plant *Miscanthus* in southern Germany (Kirchhof et al., 2001). Other potential N-fixing bacteria, such as *Azospirillum doebereinerae* (Eckert et al., 2001) and bacterial consortia consisting of N_2_-fixing clostridia (Miyamoto et al., 2004) has also been isolated from *Miscanthus*. *Herbaspirillum frisingense* strains were also recovered from other biomass grasses, *Spartina pectinata* and *Pennisetum purpureum*, grown in temperate conditions. Model calculations proposed that *Miscanthus x giganteus* gained substantial nitrogen from the N-fixation by endophytic symbionts (Davis et al., 2010), but the type of nitrogen fixers remains unclear. *H. seropedicae* isolates were shown to fix nitrogen in association with wild rice, but not with cultivated rice (Elbeltagy et al., 2001).

The entire *HsSmR1* genome (Pedrosa et al., 2011) and various other *Herbaspirillum* genomes (Table 1) from diverse habitats were recently sequenced, while that of *HrM1* was partially sequenced (Monteiro et al., 2012a). Sequenced *Herbaspirillum* species include plant growth promoting soil bacteria (*HGW103*) from the rhizosphere of the grass *Phragmites australis* (Lee et al., 2012), isolates (*HlP6-12*) from the root nodules of *Phaseolus vulgaris* (Weiss et al., 2012), strains (*HCF444* and *HYR522*) colonizing poplar (Brown et al., 2012), a strain (*HhIAM*) isolated from Japanese well water (De Souza et al., 2013) and an isolate (*HJC206*) from human fecal flora (Lagier et al., 2012).

**Table 1:** **Bacteria included in the genome/protein comparison**.

**Species**	**Abbreviation**	**Available sequences**	**Isolated from**	**Accession number**	**References**
*Herbaspirillum rubrisubalbicans* M1	*HrM1*	SSH library	Various grasses		Monteiro et al., 2012a
*Herbaspirillum huttiense subsp. putei* IAM 15032	*HhIAM*	Contigs	Well water	ANJR00000000	De Souza et al., 2013
*Herbaspirillum lusitanum* P6-12 (DSM 17154)	*HIP6-12*	Contigs	Root nodules of *Phaseolus vulgaris*	AJHH00000000	Weiss et al., 2012
*Herbaspirillum sp.* GW103	*HGW103*	Contigs 4655 proteins	Rhizosphere of *Phragmites australis*	AJVC00000000	Lee et al., 2012
*Herbaspirillum sp.* JC206	*HJC206*	Contigs	Human fecal flora	CAHF00000000	Lagier et al., 2012
*Herbaspirillum sp.* CF444	*HCF444*	Contigs 4974 proteins	Rhizosphere and endosphere of * Populus deltoide*	AKJW00000000	Brown et al., 2012
*Herbaspirillum sp.* YR522	*HYR522*	Contigs 4612 proteins	Rhizosphere and endosphere of *Populus deltoide*	AKJA00000000	Brown et al., 2012
*Herbaspirillum seropedicae* Os45	*HsOs45*	Contigs	Rice roots	AMSA00000000	Zhu et al., 2012
*Herbaspirillum seropedicae* Os34	*HsOs34*	Contigs	Rice roots	AMSB00000000	Ye et al., 2012
*Herbaspirillum seropedicae* SmR1	*HsSmR1*	Full genome 4735 proteins	Tropical grasses	CP002039	Pedrosa et al., 2011
*Herbaspirillum frisingense* GSF30^T^	*HfGSF30*	Contigs 4871 proteins	Various grasses	AEEC00000000	This work
*Gluconacetobacter diazotrophicus* PAI5	*GdPAI5*	Full genome 3851 proteins	Sugarcane	AM889285–AM889287	Bertalan et al., 2009
*Azoarcus sp.* BH72	*AzoaBH72*	Full genome 3989 proteins	Kallar grass	AM406670	Krause et al., 2006
*Klebsiella pneumoniae* 342	*Kp342*	Full genome 5768 proteins	Maize	CP000964–CP000966	Fouts et al., 2008
*Azospirillum sp.* B510	*AzospB510*	Full genome 6309 proteins	Rice	AP010946–AP0109452	Kaneko et al., 2010

Detailed descriptions of the entire genome sequences from various distant, well-described endophytes with defined endophytic habitats and plant growth promoting capabilities include *Azoarcus sp.* BH72 (*AzoaBH72*, a β-proteobacterium) (Krause et al., 2006), *Klebsiella pneumoniae* 342 (*Kp342*, γ-proteobacterium) (Fouts et al., 2008), *Azospirillum sp*. B510 (*AzospB510*, α-proteobacterium) (Kaneko et al., 2010) and *Gluconacetobacter diazotrophicus* PAl5 (*GdPAI5*, α-proteobacterium) (Bertalan et al., 2009). However, fundamental questions regarding their competitiveness, specificity to invade selected hosts, manipulate the plant growth, strategies for nutrition and survival in the plants, and the essential set of genes required for endophytic life, remain unclear.

Although it is desirable to have entire genome sequences available, the comparison of the genomic inventories does not necessarily require completely assembled genomes. Instead, comparisons of incomplete draft genome sequences with related species represents often a sufficient powerful approach for the identification of similarities and differences in their genomic inventory (Almeida et al., 2009; Studholme et al., 2009).

Here, the bacterial genome of *Herbaspirillum frisingense* GSF30^T^ was sequenced and annotated. The genome (containing a few gaps) was compared to other *Herbaspirillum* strains and selected, well-described plant endophytes. These served as references to compare the basic genome equipments necessary to colonize the endophytic niche. The lack of the type III secretion system, diversity in other secretion systems and major differences in the basic metabolic capacities characterize *Herbaspirillum frisingense* as a non-pathogenic, diazotrophic endophytic grass colonizer that is closely related to non-diazotrophic *Herbaspirillum* strains that were isolated from the rhizosphere and from well water.

## Materials and methods

### Sequencing

*H. frisingense* GSF30^T^ was grown over night at 30°C on LB-media containing 50 μg/l kanamycin. Genomic DNA was isolated and sequenced with the Roche/454 GS FLX system and with illumina technology, to increase the coverage and to close gaps. Sequencing and de-novo assembly was performed by GATC Biotech AG (Germany). The entire genome shotgun sequencing project has been deposited at DDBJ/EMBL/GenBank under the accession AEEC02000000 (Accession: PRJNA50373, ID: 50373).

### Genome annotation

Open reading frame prediction and annotation were performed by the NCBI Prokaryotic Genomes Automatic Annotation Pipeline (PGAAP) in April 2013.

### Phylogeny

16S rRNA sequences of all 14 bacteria were obtained from NCBI and analyzed with MEGA5.2 (Tamura et al., 2011). The sequences were aligned using ClustalW and the phylogeny reconstruction was done using the Maximum Likelihood method with 500 Bootstrap Replications. Marker protein sequences (or proteins predicted from draft genome sequences) were selected with AMPHORA2 (Wu and Scott, 2012). Four sequences were not identified in *HsOs45* (*rplK*, *rpoB*, *rplL*, * rplA*) and were excluded, as well as duplicate sequences. A concatenated tree and phylogenetic analysis was conducted with MEGA5.20.

### Genome comparisons

All bacteria included in the genome/protein comparison are shown in Table 1. Among these are six without protein annotation, five have draft genome information, while sufficient publically available data for comparison is lacking for *Herbaspirillum rubrisubalbicans* M1. The partial, fragmented genomic sequences available for *Herbaspirillum sp*. isolates B501, B59, and B65 were not included. *Herbaspirilla* nucleotide sequences were searched with annotated protein sequences, preferably from *Herbaspirillum seropedicae* SmR1, using NCBI's tblastn algorithm against whole-genome shotgun contigs (wgs) databases. Ambiguous hits (expect value >e-50 or identically predicted amino acids <80%), or multiple hits were reviewed with blastx against the nr protein database.

## Results

### Genome sequencing and annotation

The genome sequence of *H. frisingense* GSF30^T^ was obtained using a combined strategy with 454 pyrosequencing (Margulies et al., 2005) and illumina technology. The 454 sequencing produced more than 600000 reads with approximately 48× coverage and 265 Mb, the illumina sequencing more than 25 million reads, ca. 420 times coverage and 2.3 Gb. From these, 93 contigs (>200 bp) were assembled with a total length of ~5.4 Mb, which is in the lower range of endophyte genomes. Compared to the similarly sequenced bacterial draft genome of *Pseudomonas syringae pv. tomato T1*, a relatively large number of contig gaps was present in the *HfGSF30* draft genome. The individual inspection of the contig borders identified that repetitive sequences likely perturbed the total assembly of sequences.

**Figure 1:**
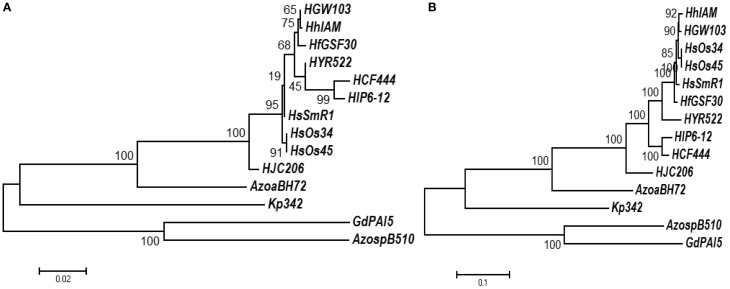
**Phylogenetic relationship based on 16S rRNA sequences (A) and marker proteins (B)**. *HfGSF30* and other *Herbaspririllum* strains and diverse endophytes. The scale bar represents 2% **(A)** or 10% **(B)** sequence divergence and numbers on the tree represent bootstrap values. *HsSmR1*, *Herbaspirillum seropedicae* SmR1; *HsOs34*, *Herbaspirillum seropedicae* Os34; *HsOs45*, *Herbaspirillum seropedicae* Os45; *HfGSF30*, *Herbaspirillum frisingense* GSF30^T^; *HGW103*, *Herbaspirillum sp*. GW103; *HhIAM*, *Herbaspirillum huttiense subsp. putei* IAM 15032; *HYR522*, *Herbaspirillum sp*. YR522; *HCF444*, *Herbaspirillum sp*. CF444; *HlP6-12*, *Herbaspirillum lusitanum* P6-12 (DSM 17154); *HJC206*, *Herbaspirillum sp*. JC206; *AzoaBH72*, *Azoarcus sp.* BH72; *AzospB510*, * Azospirillum sp*. B510; *Kp342*, *Klebsiella pneumoniae* 342; *GdPAI5*, *Gluconacetobacter diazotrophicus* PAl5.

The contig endings were manually compared using NCBI's blastn to the full genome of *Herbaspirillum seropedicae* SmR1 and a clear colinearity was identified for around 70% of the contigs, leaving 28 gaps. These sequences were used to carry out the analysis. On average, the coverage of contigs with more than 500 bp was ~400, which likely represents >99.9% of the entire sequence. The genomic inventory and its relation to distinct physiological processes is discussed below; the references for the endophyte genomes are given in Table 1. Based on their 16S rRNA sequence, *H. frisingense* is phylogenetically most closely related to *HGW103* and *HhIAM* (Figure 1A). However, based on the sequence similarity of 27 marker proteins, *HfGSF30* groups outside a cluster containing the *H. seropedicae* strains and *HGW103* and *HhIAM* (Figure 1B).

### Protein secretion systems

**Figure 2:**
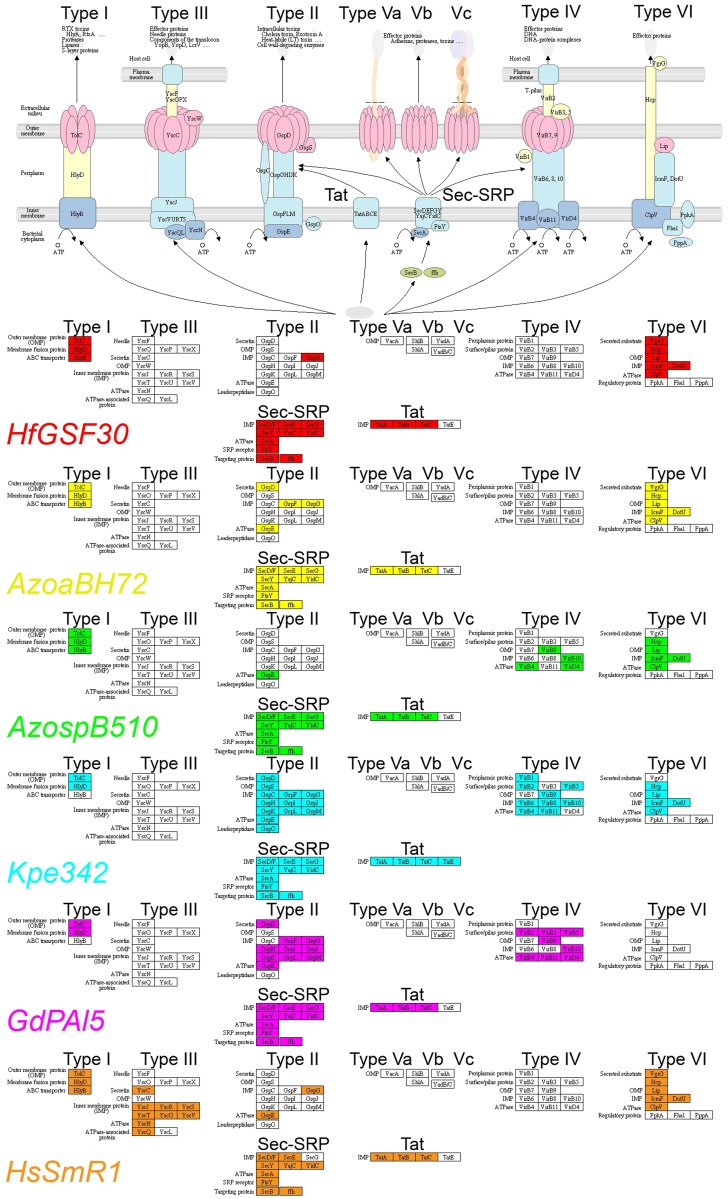
**Presence of secretion systems in *HfGSF30* and comparison with other endophytes**. *HfGSF30* genome (red), *AzoaBH72* (yellow), *AzospB510* (green), *Kp342* (blue), *GdPAI5* (pink), and *HsSmR1* (orange). Missing genes are shown in white.

**Figure 3:**
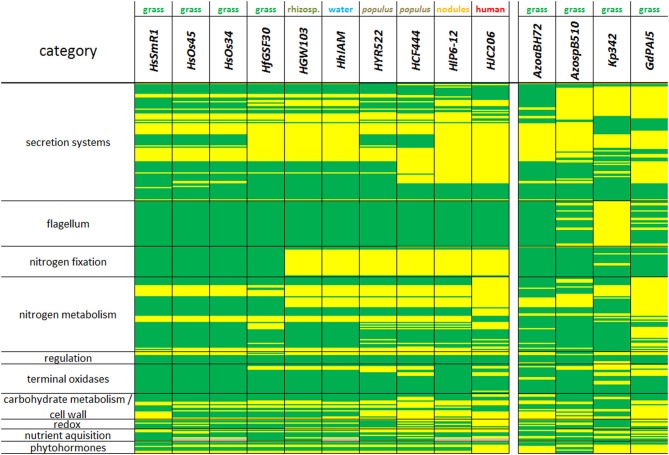
**Similarity and divergence of gene clusters reflecting various cell functions, including secretion systems, cell wall, nitrogen, carbon, and hormone metabolism in *Herbaspirillum* and endophyte strains**. Present genes are schown in green, lacking genes are shown in yellow, missing information is given in gray. Strains from left to right: *HsSmR1*, *Herbaspirillum seropedicae* SmR1; *HsOs34*, *Herbaspirillum seropedicae* Os34; *HsOs45*, * Herbaspirillum seropedicae* Os45; *HfGSF30*, *Herbaspirillum frisingense* GSF30^T^; *HGW103*, *Herbaspirillum sp*. GW103; *HhIAM*, *Herbaspirillum huttiense subsp. putei* IAM 15032; *HYR522*, *Herbaspirillum sp*. YR522; *HCF444*, *Herbaspirillum sp*. CF444; *HlP6-12*, *Herbaspirillum lusitanum* P6-12 (DSM 17154); *HJC206*, *Herbaspirillum sp*. JC206; *AzoaBH72*, *Azoarcus sp.* BH72; *AzospB510*, *Azospirillum sp*. B510; *Kp342*, *Klebsiella pneumoniae* 342; *GdPAI5*, *Gluconacetobacter diazotrophicus* PAl5.

The *HfGSF30* genome encodes the type I, type VI, Sec-SRP and the Tat (twin-arginine translocation) systems, but lacks the type III secretion system, as shown in Figure 2. The type III secretion system is typically used by pathogenic bacteria to deliver effector proteins into the plant host cells, but is also used in beneficial interactions for optimization (Viprey et al., 1998). *HfGSF30*, as well as the reference grass endophytes *AzoaBH72*, *AzospB510*, *Kp342*, and *GdPAI5*, completely lack the type III secretion system *hrp*/*hrc* genes (Figures 2, 3, Table S1). By contrast, other *Herbaspirillum* grass endophytes and poplar colonizers, namely *HsSmR1*, *H. rubrisubalbicans* M1, *HsOs34*, *HsOs45*, *HCF444*, and *HYR522*, contained that system. It is critical for pathogenicity, but also endophytic invasion of *HrM1* (Monteiro et al., 2012a). Pedrosa et al. (2011) found no transposon elements flanking the type III secretion system genes in *HsSmR1*, suggesting that it was not recently added into the genome. Flanking regions of the type III secretion system genes were only partially conserved among *Herbaspirillum* strains, suggesting that the type III protein secretion was deleted in some *Herbaspirillum* strains, including *HfGSF30*.

All *Herbaspirillum* strains lack the type IV secretion system, which is involved in virulence and horizontal gene transfer (Juhas et al., 2008), but genes of this system are identified in more distant endophytes, such as *AzospB510*, *Kp342*, and *GdPAI5* (Figures 2, 3, Table S1). Except for *Kp342*, all mentioned endophytes and *Herbaspirillum* strains contain the entire machinery for flagellum export and function (Figure 3).

Furthermore, a reduced set of the type IV pilin secretion/fimbrial assembly genes, members of the type II secretion system, was identified in *HfGSF30* (similar as in *HGW103* and *HhIAM*), when compared to *H. seropedicae* strains. The tree colonizers *HCF444* and *HYR522* have almost the same set of type IV pilin secretion genes as *HsSmR1*. These genes were completely absent in *AzospB510*, *Kp342*, and *GdPAI5*, while they were present in *AzoaBH72*.

*HfGSF30* possesses type VI secretion system genes. This system is involved in host-bacteria interaction, both in pathogenic and symbiotic relationships (Filloux a Hachani and Bleves, 2008). These genes are also present in most *Herbaspirillum* strains and all considered grass endophytes, except *GdPAI5*. Notably, the type VI system is present in one, but lacking in another *Herbaspirillum* strain isolated from poplar, and is also absent the strains isolated from nodules (*HIP6-12*) and from human fecal flora (*HJC206*). *HfGSF30* contains the chaperone-usher system (type I pilus assembly proteins), whereas some *Herbaspirillum* strains, including *H. seropedicae* isolated from rice, and only *Kp342*, but not *AzoaBH72*, *AzospB510*, and *GdPAI5*, contain that system (Figure 3, Table S1).

### Nitrogen metabolism

The acetylene reduction assay has suggested nitrogenase activity in *HfGSF30* (Kirchhof et al., 2001). Among the *Herbaspirillum* strains, nitrogen fixation genes were only present in *H. seropedicae* strains and *HfGSF30*. The *nif*-region is very similar to the corresponding region of *H. seropedicae* SmR1 with 94% nucleotide identity, 96% amino acid identity and identical gene arrangement. Some gene products, nifB, nifX, nifZ1, fdxB, and fix, were even 100% identical between *HfGSF30* and *HsSmR1*. *Nif* genes are absent in *HGW103*, *HhIAM*, *HYR522*, *HCF444*, *HJC206*, and even in *HlP6-12*, which was isolated from *Phaseolus* nodules. The *AzospB510*, *AzoaBH72*, *GdPAI5*, and *Kp342* grass endophytes contain the entire *nif* cluster (Figure 3).

*HfGSF30* is equipped with an assimilatory nitrate reductase (*nasAC*) and a NAD(P)H-dependent nitrite reductase (*nirBD*; EC 1.7.1.4), similar to *AzospB510*, *AzoaBH72*, *Kp342* and other *Herbaspirillum* strains, except for *HJC206* (and *GdPAI5)*, which completely lack nitrate assimilatory and dissimilartory genes (Figure 3). *HfGSF30*, *HsSmR1*, *HsOs34*, and *HsOs45* strains contain the respiratory nitrate reductase (*narGHJI*), the nitrite/nitrate transporter (*narU*) and a nitrate/nitrite sensor histidine kinase transcription regulator (*narXL*) to utilize nitrate in anaerobic respiration. *Kp342* has a similar set of genes, but other *Herbaspirillum* isolates, *AzoaBH72*, *GdPAI5*, and *AzospB510* apparently cannot utilize nitrate as alternative electron acceptor in anaerobic conditions. The absence of nitrate reductase in *HJC206* is consistent with the minor role of nitrate in the human habitat (Lagier et al., 2012), but the endophyte *GdPAI5* also lacks the respective genes. The presence of α-, δ- and γ-subunits of a formate dehydrogenase (EC 1.2.1.2) parallels the occurrence of genes for nitrate reduction, and is absent in *GdPAI5* and *HJC206*. However, *HJC206* has formate dehydrogenase genes with sequence similarity to *Herminiimonas arsenicoxydans* that are unique in the *Herbaspirillum* genus.

*HfGSF30* is likely capable to reduce nitrate to NO and further to N_2_O (EC 1.7.2.1, 1.7.99.7), a feature exclusively present in *HfGSF30* among *Herbaspirillum* strains, but no nitrous oxide reductase (EC 1.7.99.6) to reduce N_2_O to N_2_ is identified (Figure 3). This is in line with previous experimental evidence, which showed that NO^−^_3_ reduction to N_2_ did not occur in *HfGSF30* (Kirchhof et al., 2001). Nitrogen reduction varies greatly in other diazotrophic endophytes, namely *AzoaBH72* appears capable to reduce NO via N_2_O to N_2_, but a nitrite reductase is missing. *GdPAI5* also lacks nitrate reductase (Cavalcante and Dobereiner, 1988). *AzospB510*, like *HfGSF30*, has the possibility to reduce NO_2_ to N_2_O, but not to N_2_.

Amino acids, such as asparagine and aspartic acid, were utilized as nitrogen sources by *HfGSF30* (Kirchhof et al., 2001), but the capabilities to synthesize aspartic acid and asparagine differ among *Herbaspirillum* strains, with only *HsSmR1* and *HJC206* containing an asparagine synthase gene (Table S1). Although the full urea cycle is present in all strains (except for *GdPAI5*), differences are identified with respect to the alternative urea degradation pathway, which is partially missing in *HfGSF30*, although it is present in all other grass endophytes.

### Respiration

*HfGSF30* contains four terminal oxidases that allow adaptation to different oxygen levels and microhabitats: cytochrome aa3 (*coxAB*); cytochrome bd-type quinol oxidase (*cydAB*), cbb3-type cytochrome c oxidase (*fixPON*), cytochrome o ubiquinol oxidase (*cyoABC*). Genes for NADH dehydrogenase, succinate dehydrogenase and cytochrome c reductase are ubiquitously identified in all *Herbaspirillum* strains.

The high affinity cbb3-type cytochrome c oxidase may support ATP-synthesis under oxygen limitation during nitrogen fixation and accordingly, this system is lacking in the non-diazotrophic *HCF444*. However, the diazotrophic *Kp342* and *GdPAI5* also lack this oxidase and it is present in other nitrogenase-lacking *Herbaspirillum* strains. Multiple *coxAB* copies are only present in *H. seropedicae* strains and in the strain isolated from nodules (*HIP6-12*). The cytochrome bd-type quinol oxidase is absent in *HCF444* isolated from poplar and *Kp342*.

### Carbohydrate metabolism and cell wall degradation

A broad spectrum of monosaccharides, organic acids and alcohols, but not di-and tri-saccharides, are utilized as carbon sources by *HfGSF30* (Kirchhof et al., 2001). This is in line with the identification of metabolizing enzymes for these substrates. *HfGSF30* lacks the sucrose-degrading enzyme invertase (EC:3.2.1.26) and α-glucosidase (EC:3.2.1.20), while *HsSmR1*, *HsOs34*, *HsOs45*, *HCF444*, *HhIAM*, and *AzoaBH72* encode α-glucosidase. *AzospB510* and *GdPAI5* lack both enzymes, while both are present in *Kp342*. Except for *HCF444* and *HJC206*, all *Herbaspirillum* strains had two trehalose synthesis pathways (*otsAB* and *treXYZ*). A gene related to the large ribulose-1,5-bisphosphate carboxylase/oxygenase (RubisCO) subunit from plants was also present in several *Herbaspirillum* strains, including *HfGSF30*, but no sequence encoding a phosphoribulokinase was found (but present in *AzoaBH72* and *Kpe342*). Therefore, CO_2_ fixation appears to be impossible for these endophytes and the RubisCO-like proteins are probably involved in sulfur metabolism (Tabita et al., 2007). A few membrane transporters were notably different within the *Herbaspirillum* strains: the arsenite/antimonite transporter was only present in *HfGSF30*, *HsOs34*, *HsOs45*, *HGW103*, and *HhIAM*, differences were also obvious in the number and type of ammonium and iron transporters (Table S1).

There is no evidence that the plant cell wall was affected by *H. frisingense* colonization (Rothballer et al., 2008), but *HfGSF30* (and *HsOs34*, *HsOs45*, *HGW103*, *HhIAM*, *HYR522*, and other endophytes) are equipped with an endo-1,4-D-glucanase that may break down cellulose (EC:3.2.1.4, absent in *HsSmR1*). Two chitin deacetylases (EC:3.5.1.41 and 3.1.1.58) are present in all *Herbaspirillum* strains, while *HsSmR1* and *HJC206* possess two additional enzymes. α-glucosidase (EC:3.2.1.20) and α –amylase (EC:3.2.1.1) are absent in *HfGSF30* and some *Herbaspirillum* strains. In *HrM1*, a large operon involved in cellulose synthesis (or degradation) appears crucial for colonization (Monteiro et al., 2012a); this entire operon was present in *HfGSF30*, *HsOs34*, *HsOs45*, *HGW103*, *HhIAM* (and *Kp342*), but was absent in *HsSmR1*, *HlP6-12*, *HYR522*, *HCF444*, *HJC206.*

### Survival against the plant defense and environmental stress

The plant defense against bacterial, fungal and viral threats generally involves the production of reactive oxygen species (ROS), nitric oxide and phytoalexins. It has recently been shown that antioxidant enzymes are up-regulated during biological nitrogen fixation to prevent ROS in *G. diazotrophicus* PAl5 (Alquéres et al., 2010), but compared to the other bacteria under study, this strain, together with the human isolate *HJC206*, contains the least number of potential detoxification genes. Different strategies to cope with reactive oxygen are apparent within *Herbaspirillum* strains and other endophytes (Figure 3, Table S1).

### Biosynthesis of plant hormones

The production of plant hormones, or other beneficial agents, is a common strategy of endophytes to promote plant growth (Hardoim et al., 2008). All *Herbaspirillum* strains, except for *HJC206*, contain the genes for 1-aminocyclopropane-1-carboxylate (ACC) deaminase to degrade the ethylene precursor ACC to 2-oxobutanoate and NH_3_. ACC is taken up by *H. frisingense* (Rothballer et al., 2008) and its efficient breakdown by ACC deaminase may reduce locally plant ethylene levels at sites of invasion (Hardoim et al., 2008). The endophytes *AzoaBH72*, *Kp342* and *GdPAI5* do not contain ACC deaminase and thus appear not capable of modulating plant ethylene signaling.

**Figure 4:**
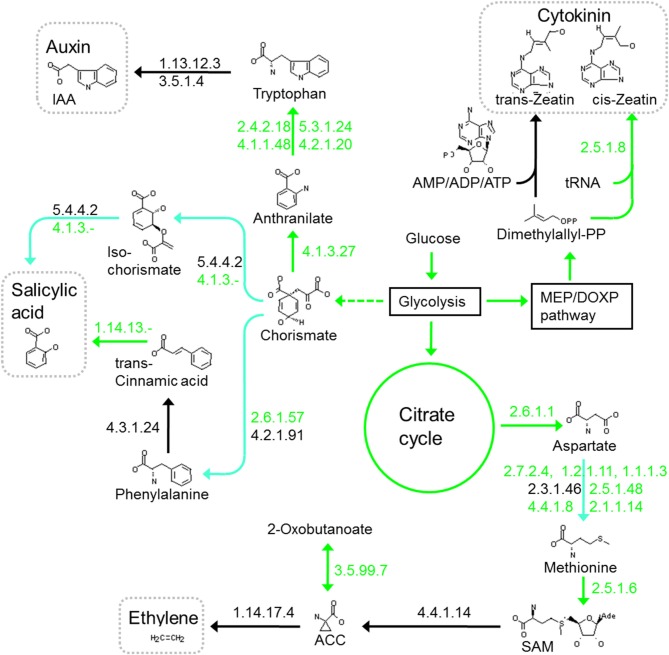
**Biosynthesis and manipulation of plant hormones in *HfGSF30***. Green arrows or EC numbers indicate possible reactions or present enzymes, turquoise arrows indicate that at least one enzyme is missing in the pathway, while black arrows or EC numbers indicate that this pathway or enzyme is missing.

Auxin (indole acetic acid) synthesis proceeds via several pathways, which are at least partially present in all *Herbaspirillum* and other grass endophytes. Differences in auxin production are suggested in the *Herbaspirillum* strains, as only *HsSmR1* encodes the amidase that releases NH_3_ and indole acetic acid from indole-3-acetamide *iaaH* (and *AzoaBH72*) (Costacurta and Vanderleyden, 1995). However, the essential tryptophan 2-monooxygenase (*iaaM*) for decarboxylation of tryptophan to indole-3-acetamide is not unambiguously identified in any *Herbaspirillum*. All *Herbaspirillum* strains lack an *ipdC* homolog, which is present in *Kp342*, where indole acetic acid may be synthesized by indole-3-pyruvate decarboxylase from tryptophan via indole-3-pyruvic acid. *Herbaspirillum* strains also lack enzymes for the indole-3-acetonitrile pathway. Tryptophan-independent reactions from indoles to indole acetic acid via transferases are likely and potential genes are abundant, but no gene appears to encode a clearcut prototype indole acetic acid-producing enzyme (Figure 4).

Lactoserines are utilized by *AzospB510* for quorum-sensing, modulate the rhizosphere density competence and the adaptation of the bacteria to the environment. *H. frisingense* GSF30^T^ failed to produce acyl homoserine lactones (Rothballer et al., 2008). In accordance with these experimental findings, the genes related to acyl homoserine lactone synthase and acylase were absent in all *Herbaspirillum* strains (Figure 3).

## Discussion

The comparison of the draft genome sequence of *HfGSF30* with the genetic inventory of related *Herbaspirillum* strains and more distant diazotrophic grass endophytes revealed a high diversity in their potential capabilities. The well-characterized endophyte *H. seropedicae* SmR1, which is associated with gramineous species like sorghum, sugarcane, rice and maize (Kirchhof et al., 2001) in warm climates, shares high nucleotide sequence identity with *HfGSF30*. However, even higher conservation in the genomic equipment was detected with non-diazotrophic *Herbaspirillum* strains that were not isolated as endophytes, but rather from the rhizosphere of Australian *phragmites* (*HGW103*) and well water (*HhIAM*).

Among the sequenced endophytes, a differential inventory for the nitrogen metabolism is striking. This suggests that a range of different metabolic capabilities allows endophytic colonization of various plant habitats, even within a single plant species. *HfGSF30* is closer related to *Herbaspirillum seropedicae* isolates from rice than to *HsSmR1*, and among more distant endophytes its metabolic capabilities most closely resemble that of *AzoaBH72*, but it has little overlap with the metabolic equipment of the sugarcane-associated *GdPAI5*. Endophytes may colonize different niches within the same plant and interact; despite their contrasting metabolic inventory, different endophytic strains were abundant in sugar cane fields that were inoculated with a bacterial inoculation mixture including *Gluconacetobacter diazotrophicus* PAI5 and *Herbaspirillum* (Fischer et al., 2012). Interestingly, even bacteria not present in the inoculum were associated with these sugarcane plants (Fischer et al., 2012).

The metabolic traits discussed above differ widely in the *Herbaspirillum* genus, in accordance with diverse habitats, manifested, e.g., by the human isolate *HJC206* or the nodule isolate *HlP6-12*. These two bacteria show least overlapping genomic capabilities with *Herbaspirillum seropedicae* strains (Figure 3). With the exception of the *Herbaspirillum* strain isolated from human fecal flora, all *Herbaspirillum* strains are equipped to utilize nitrate as a nutrient and reduce it to ammonium. This is not a common feature of plant endophytes, as *GdPAI5* lacks all essential nitrate assimilation genes. The capability of anaerobic respiration using nitrate as an electron acceptor in *HfGSF30*, *HsSmR1*, *HsOs45*, and *HsOs34* correlates with the presence of nitrogen fixation genes, suggesting that these strains can adapt to low nitrogen and oxygen availabilities. This is also underscored by the tendency that these strains have higher number of terminal oxidase genes.

*H*. *frisingense* GSF30^T^ turned out unique as a potential N_2_O producer among the *Herbaspirillum* strains. Significant N_2_O emissions, exceeding those of a heavily fertilized rye field, but less than those from fertilized maize, have been reported from fertilized *M. x giganteus*, a host of *HfGSF30* (Jørgensen et al., 1997; Gauder et al., 2012). However, not relevant N_2_O emissions were detected from unfertilized *M. x giganteus* (Jørgensen et al., 1997; Gauder et al., 2012).

Hormone production and/or degradation may contribute to the variable growth promoting effect of *Herbaspirillum* strains. The metabolic pathways that produce these metabolites have been identified by analytical tests (Rothballer et al., 2008) and in the sequence. *AzoaBH72*, a native colonizer of Kallar grass, appears in many aspects similar to *HfGSF30*. For example, both strains lack the entire type IV secretion system, which is partially present in the other sequenced endophyte genomes, but not in the *Herbaspirillum* genus. Highly relevant is the lack of the type III system in *HfGSF30* (and in *HhIAM*, *HlP6-12*, *HGW103*, *HJC206*), its presence and importance for colonization in *HsSmR1* (and *HsOs45*, *HsOs34*, *HCF444*, *HYR522*) and *HrM1* (Monteiro et al., 2012a); and similar diversity within parts of the type II system. The different sets of secretion systems in *HfGSF30* are compatible with the observed broad host ranges and no pathogenicity associated with this strain. Furthermore, several further candidate genes that are potentially involved in plant colonization, e.g., genes encoding attachment proteins of the hemagglutinin-type and genes involved in lipopolysaccharide formation and export differ between individual *Herbaspirillum* strains (Monteiro et al., 2012a). The absence of flagella that often harbor molecular patterns that are recognized by the plant pathogen defense, may be an advantage for high colonization numbers by *Kp342* (Fouts et al., 2008). However, *HfGSF30* and the other endophytes contain the entire flagella machinery, and this suggests that the flagellum plays an important role for these organisms, similar as in other root colonizing bacteria. For example, in *Azospirillum brasilense* Sp7, the flagellum is not only crucial for the chemotactic movement toward the root, but also for the initial attachment and final anchoring to the root surface. Mutants impaired in flagella formation are severely hampered in their colonization efficiency (Croes et al., 1993). However, it is also known that in contact with the plant, *Azospirillum brasilense* strains undergo substantial pleomorphic changes which also includes the loss of the flagellum (Assmus et al., 1995).

In summary, the *HfGSF30* genome shows high similarity to the well known diazotrophic endophyte *Herbaspirillum seropedicae*, but even higher similarity (except for nitrogen fixation) with genomes from strains isolated from Australian *phragmites* rhizosphere and Japanese well water. High similarity in secretion systems and cell wall metabolism, among other traits, may suggest that either the respective habitats of these *Herbaspirillum* strains (*HfGSF30*, *HGW103*, *HhIAM*) are wider or that minor differences can confer different habitat competence. Grass endophytes do not only utilize highly diverse interaction (secretion) and attachment systems, but individual endophytes utilize highly different basic metabolic modules to survive in grasses. Endophytic, rhizosphere-competent and well water *Herbaspirillum* bacteria have surprising overlap in their genomic equipment.

## Author contributions

Daniel Straub carried out all sequence annotations and molecular genetic analysis, Michael Rothballer, Anton Hartmann and Uwe Ludewig participated in the analysis and writing of the manuscript. Uwe Ludewig designed the study, and all authors read and approved the final manuscript.

## Acknowledgments and funding

We thank Dr. Marek Dynowski (University of Tübingen, Germany) for software advice, the Deutsche Forschungsgemein- schaft and EU grant “Biofector” for partial financial support and Prof. Ralf Kaldenhoff (Technical University of Darmstadt, Germany) for generous support. We thank S. Demyan for proof reading.

### Conflict of interest statement

The authors declare that the research was conducted in the absence of any commercial or financial relationships that could be construed as a potential conflict of interest.

## Abbreviations

EC: Enzyme classification
*AzoaBH72*: *Azoarcus sp.* BH72
*Kp342*: *Klebsiella pneumoniae* 342
*AzospB510*: *Azospirillum sp*. B510
*GdPAI5*: *Gluconacetobacter diazotrophicus* PAl5
*HfGSF30*: *Herbaspirillum frisingense* GSF30^T^
*HsSmR1*: *Herbaspirillum seropedicae* SmR1
*HrM1*: *Herbaspirillum rubrisubalbicans* M1
*HhIAM*: *Herbaspirillum huttiense subsp. putei* IAM 15032
*HlP6-12*: *Herbaspirillum lusitanum* P6-12 (DSM 17154)
*HGW103*: *Herbaspirillum sp*. GW103
*HJC206*: *Herbaspirillum sp*. JC206
*HCF444*: *Herbaspirillum sp*. CF444
*HYR522*: *Herbaspirillum sp*. YR522
*HsOs34*: *Herbaspirillum seropedicae* Os34
*HsOs45*: *Herbaspirillum seropedicae* Os45.
